# Microhabitat locality allows multi-species coexistence in terrestrial plant communities

**DOI:** 10.1038/srep15376

**Published:** 2015-10-20

**Authors:** Jerrold M. Tubay, Keisuke Suzuki, Takashi Uehara, Satoshi Kakishima, Hiromu Ito, Atsushi Ishida, Katsuhiko Yoshida, Shigeta Mori, Jomar F. Rabajante, Satoru Morita, Masayuki Yokozawa, Jin Yoshimura

**Affiliations:** 1 Graduate School of Science and Technology, Shizuoka University, Hamamatsu 432-8561, Japan; 2 Mathematics Division, Institute of Mathematical Sciences and Physics, University of Philippines Los Baños, College, Laguna 4031, Philippines; 3 Department of Mathematical and Systems Engineering, Shizuoka University, Hamamatsu 432-8561, Japan; 4 Department of Preschool Education, Nagoya College, Toyoake, Aichi 470-1193, Japan; 5 Center for Ecological Research, Kyoto University, Otsu, 520-2113, Japan; 6 Center for Environmental Biology and Ecosystem Studies, National Institute for Environmental Studies, Tsukuba, 305-8506, Japan; 7 Faculty of Agriculture, Yamagata University, Tsuruoka, Yamagata 997-8555, Japan; 8 Marine Biosystems Research Center, Chiba University, Kamogawa, Chiba 299-5502, Japan; 9 Department of Environmental and Forest Biology, State University of New York College of Environmental Science and Forestry, Syracuse, NY 13210, USA

## Abstract

Most terrestrial plant communities exhibit relatively high species diversity and many competitive species are ubiquitous. Many theoretical studies have been carried out to investigate the coexistence of a few competitive species and in most cases they suggest competitive exclusion. Theoretical studies have revealed that coexistence of even three or four species can be extremely difficult. It has been suggested that the coexistence of many species has been achieved by the fine differences in suitable microhabitats for each species, attributing to niche-separation. So far there is no explicit demonstration of such a coexistence in mathematical and simulation studies. Here we built a simple lattice Lotka-Volterra model of competition by incorporating the minute differences of suitable microhabitats for many species. By applying the site variations in species-specific settlement rates of a seedling, we achieved the coexistence of more than 10 species. This result indicates that competition between many species is avoided by the spatial variations in species-specific microhabitats. Our results demonstrate that coexistence of many species becomes possible by the minute differences in microhabitats. This mechanism should be applicable to many vegetation types, such as temperate forests and grasslands.

Almost all terrestrial plant communities usually consist of many species and beside some climax forests, natural communities with a single species are rare^1^,^2^,^3^. This suggests that the coexistence of many plant species is a common and ubiquitous feature of natural communities. However, all these coexisting species are in fact competing for the same resources, e.g., light, soil nutrients and water^3^,^4^,^5^,^6^,^7^,^8^. Here we consider the mechanism that allows for the coexistence of competing species in terrestrial vegetation such as temperate forests and grasslands. The famous niche theory suggests that the coexistence of competing species becomes possible only if their niches are distinctive, due to the avoidance of competition between species^3^,^9^,^10^,^11^. Previous mathematical studies show that the coexistence of competing species is difficult unless some factors promoting coexistence, such as symbiosis, specific tradeoffs, and extrinsic factors, are considered^1^,^2^,^3^. However, even in forests and grasslands where no such factors were recorded, we still find high species diversity^11^,^12^,^13^. In these communities, environmental factors and conditions are fairly even except for minute differences in microhabitats, and Tilman suggested that such micro-environmental variations can be responsible for the coexistence tree species in temperate forests^1^,^2^. For instance, two small adjacent patches of soil can be distinctive in terms of composition resulting to different establishment rates for each plant species. Using a tree-based simulation model, Takenaka showed that the diversity of tree species is clearly affected by seedling establishment rate^14^. However, Takenaka’s model is an example of a lottery model^15^,^16^,^17^,^18^,^19^ that will result to competitive exclusion if taken to sufficiently long time frames while our model can maintain stable coexistence given the same time frame, which is the major difference between our studies. Takenaka’s model is in line with prominent studies relating spatial heterogeneity and coexistence such as those of Chesson, Muko and Iwasa which are mostly based on a lottery model for sessile organisms^16^,^17^,^18^,^19^. As stated previously, these models can only maintain coexistence in shorter periods and only for few species. Moreover, they often have tractability issues because of the many factors and parameters involved.

In this paper, we introduce the spatial heterogeneity in microhabitats between species in a simple lattice model: a lattice Lotka-Volterra competition model. It is also called a multi-species contact process. The soil system of terrestrial communities is extremely variable^20^. Two soil samples 5 cm apart are usually very different in water contents, nutrient conditions and other factors because of the litter differences in dead leaves, debris, animal carcasses, and so on^12^. To represent the spatial heterogeneity in soil microhabitats, we assign the settlement rates of species randomly over the entire lattice space. The settlement rate here represents the germination and seedling success of a seed that landed on a lattice site. We also used the multiple contact process to explain the paradox of enrichment of the phytoplankton by induced competitive interaction^15^. However, the methodology in this study is different since spatial heterogeneity in freshwater ecosystems like lakes and ponds is often much lower than in the soil.

We show that more than 10 species are generally plausible to coexist in a small lattice space. The number of coexisting species increases more than 15 species, when species-specific heterogeneity is introduced in the mortality rates of matured (settled) plants. This is in contrast with Muko and Iwasa finding that spatial variation in mortality only leads to coexistence, not in fecundity^19^. In this study, coexistence is enabled by minute variation in both fecundity and mortality independently, or combined. This coexistence dynamics should be applicable to temperate forests, grasslands and other vegetation types. We will also discuss the general mechanism for species coexistence in animal and plant communities from the spatial heterogeneity of microhabitats or minute niches.

## Results

**Figure 1:**
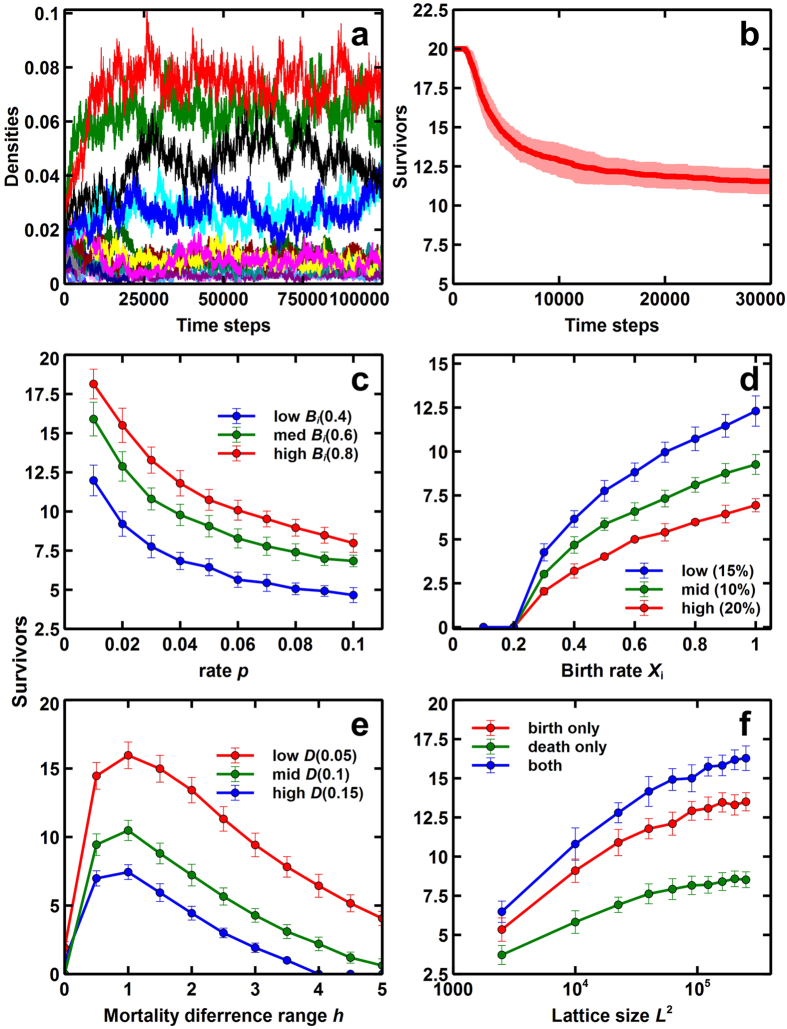
Population dynamics of 20-species community in a lattice Lotka-Volterra competition model with species- and site-specific variability. (**a**) Temporal density dynamics with the variable birth rates. (**b**) Temporal changes in the number of surviving species (Average of 50 runs with the transparent area as deviations). (**c**) The effect of fecundity *B*_*i*_ on the number of surviving species. (Average of 50 runs at 30,000 steps). (**d**) The effect of difference of birth rate on the number of surviving species (Average of 50 runs at 30,000 steps with deviations). (**e**) The effect of mortality range *h* (=0 ~ 5) on the number of surviving species (Average of 50 runs at 30,000 steps with deviations). (**f**) The effect of lattice size (*L* × *L*) on the number of surviving species (Average of 50 runs at 30,000 steps with deviations). The parameter conditions are as follows: Lattice size is 200 × 200, except (**f**). Birth rates are *B*_*i*_ = *a* − (*i* − 1)*r*, where *a* = 0.8, *r* = 0.002 unless specified (**c**) *a* = 0.8 (red), *a* = 0.6 (green), *a* = 0.4 (blue); (**d**) *p* = 0.05 (blue), *p* = 0.01 (red), *p* = 0.02 (green), where 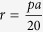; (**e**) *D* = 0.05 (red), *D* = 0.1 (green), *D* = 0.15 (blue); (**f**) both: *D* = 0.05 (red), birth only: *D* = 0.1 (green), and death only: *D* = 0.05 (blue). The random variables *ε*_*i*_[*m*, *n*] and *δ*_*i*_[*m*, *n*] follow a standard uniform distribution over 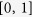.

In the current simulation of 20 initial species in a lattice Lotka-Volterra competition model, more than 10 species persisted with species- and site-specific variability in birth rates. Temporal dynamics indicated that each species fluctuated over time but species with higher average birth rates tend to keep higher average densities (Fig. 1a, S1 and S2a–c). The number of surviving species declined rapidly in the initial steps, but soon stopped decreasing, resulting in about 12 coexisting species (Fig. 1b). The number of surviving species increases with the reduction in the in the upper bound *p* (Fig. 1c) and with the increase in the basic birth rates *a* (Fig. 1d). Unlike the results in the lattice model without species- and site-specificity in phytoplankton^15^, increasing birth rates does not induce enough competition that can result to exclusion since microhabitat differences has a niche-separation effect. In the extended model, the species-specific variability in mortality also enhances diversity (Fig. 1e,d). Here, we can observe that the number of surviving species increases with the reduction in the basal mortality rate *D* and an optimum exists between the mortality difference ranges *h*. The former is expected though the latter was unforeseen. This is because an increase in *h* leads to a higher mortality rate which can possibly lead to a steady decrease in total species survival. Conversely, the total number of species is enhanced by combining the local variability of both reproduction and mortality, and by increasing the carrying capacity (lattice size *L*) (Fig. 1f).

**Figure 2:**
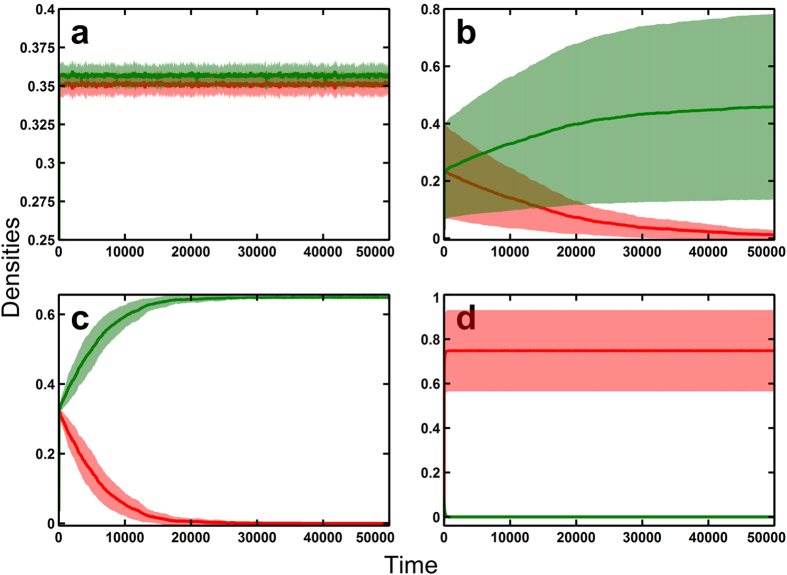
Temporal dynamics of 2 species in a lattice Lotka-Volterra competition model with species- and site-specific variability in birth rates and its controls (50,000 steps with an average of 50 runs). The transparent area are the deviations. (**a**) With species- and site-specific variability. (**b**) With no variability. (**c**) With site-specific variability only. (**d**) With species-specific variability only. (**b–d**) are various controls. Birth rates are all *R*_*i*_ = 0.8 − 0.002(*i* − 1) (Red species has higher birth rate), Death rates are *D*_*i*_ = 0.1 (All species have same rate), lattice size are *L* × *L* = 200 × 200 and the local settlement rate *ε*_*i*_[*m*, *n*] follows a standard uniform distribution over [0, 1] with 32,767 divisions.

In order to elucidate the underlying mechanisms of coexistence, we investigate the lattice model with two-species systems. We compared the current model with various controls: (1) The model with species/site variability; (2) Control 1 with no variability; (3) Control 2 with site variability only; and (4) Control 3 with species variability only (Table 1). The model and controls were run 30,000 steps on the average of 50 simulation runs. The figures show the averages and deviations (transparent area) of the densities from the simulations (Fig. 2). While the current model exhibits the stable coexistence of two species with very little deviations (Fig. 2a and S3a,b), all three controls exhibits the exclusion of one species for all simulation runs (Fig. 2b–d and S3d–j). Thus, species-variability or site-variability alone is not sufficient for the coexistence of two species, indicating that the simultaneous species-specific microhabitat variability is the key for multiple-species coexistence.

**Figure 3:**
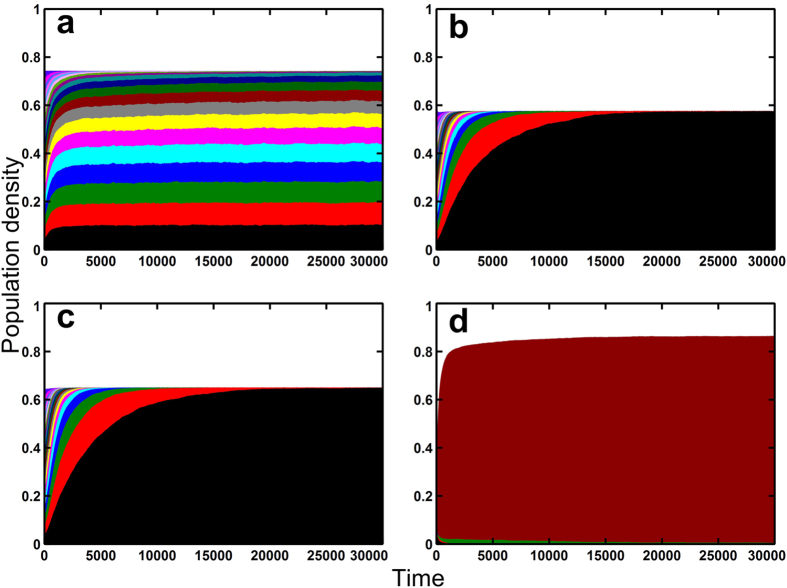
Average local dynamics of 20 species in a lattice Lotka-Volterra competition model with species- and site-specific variability in birth rates and its controls (50,000 steps with an average of 50 runs). The transparent area are the deviations. (**a**) With species- and site-specific variability. (**b**) With no variability. (**c**) With site-specific variability only. (**d**) With species-specific variability only. (**b–d**) are various controls. Birth rates are all *R*_*i*_ = 0.8 − 0.002(*i* − 1) (Red species has higher birth rate), Death rates are *D*_*i*_ = 0.1 (All species have same rate), lattice size are *L* × *L* = 200 × 200 and the local settlement rate *ε*_*i*_[*m*, *n*] follows a standard uniform distribution over 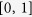 with 32,767 divisions.

This elucidation is further developed with a 20-species system with similar controls as in the 2-species system, respectively. These simulations account for sensitivity with the increase in the number of species. We stacked the average densities of 50 runs through 50,000 steps so that coexistence and exclusion can be clearly observed. Figure 3a, which represents the model visibly shows coexistence of 20 species. This figure shows more species coexistence compared to that in Fig. 1a since a lower mortality rate was used for this 20-species system. Although Fig. 2b,c show an average of 2 species coexisting after 50,000 steps, the trend shows that both figures will eventually lead to a single-species exclusion. Like the 2-species system (Fig. 2), it is apparent that without species- and site-variability, exclusion is likewise inevitable for the 20-species system (Fig. 3a).

**Figure 4:**
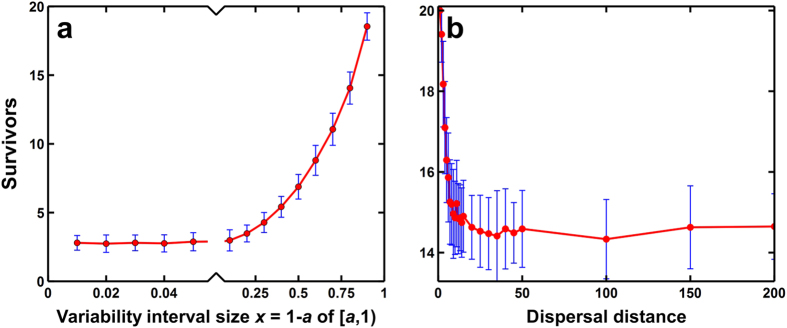
The effect of variability range for *ε*_*i*_[*m*, *n*] (local dynamics) and dispersal distance to diversity (50,000 steps with an average of 50 runs including deviations). (**a**) By default, *ε*_*i*_[*m*, *n*] follows the uniform distribution over 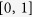. This is the effect of reducing the size of the interval (*a*, 1) where *ε*_*i*_[*m*, *n*] is defined with 0 < *a* < 1. (**b**) Simulations were conducted for each dispersal distance starting local dispersion where parents can only disperse its seeds to adjacent sites (1 square lattice around) to global dispersion.

Additionally, we looked at the effects of the range of variability and seed dispersal to plant diversity. By default, 0 ≤ *ε*_*i*_[*m*, *n*] ≤ 1. By increasing the lower bound for *ε*_*i*_[*m*, *n*] (e.g., *a* ≤ *ε*_*i*_[*m*, *n*] ≤ 1 where 0 < *a* < 1), we reduce the range of values by which the specificity/variability *ε*_*i*_[*m*, *n*] is defined. As anticipated, decreasing the interval size where *ε* is obtained decreased the number of surviving species (Fig. 4a). However, it can be noted that even minute species- and site-specific variability range results to coexistence which is approximately 3 species when 0.99 ≤ *ε*_*i*_[*m*, *n*] ≤ 1. In Fig. 4b, plant diversity is determined with each increase in dispersal distance measured in lattice squares. Results show that increasing dispersal distance decreases plant diversity (Fig. 4b). In fact, we can maintain the initial number of 20 species without any species dying out after 20,000 time steps (Fig. 4b and S2) at dispersal distance of one square around the parent. Limiting the dispersal distance enhances species coexistence.

## Discussion

Our results demonstrate that species- and site-specific variability in seedling survival guarantees the coexistence of multiple species. In this system, the number of coexisting species increases with basic fecundity *B*_*i*_, suggesting that the survival of weaker species is affected easily by fecundity (Fig. 1c,d). On the other hand, if the difference *p* in birth rate between the strongest and weakest species is decreased, the surviving species increase because all species becomes similar (Fig. 1c,d). Thus, the species and site heterogeneities in microhabitats guarantee the coexistence of multiple plant species.

One might argue that site-specific heterogeneity will logically promote diversity. However, based on the temporal dynamics examined in a 2-species lattice system (and 20-species system), this is not the case (Figs 2c and 3c). Clearly, site variability alone does not lead to species diversity, surely not in two- and 20-species lattice systems. It is clear from the simulations that both species- and site-specific heterogeneities are needed to promote species coexistence. Moreover, no matter how minute, small variability is better for coexistence than none at all (Figs 2b, 3b and 4a).

The tree-based simulation model showed that forest coexistence between trees is directly affected by seed establishment rate^14^. Though his model is a good representation of coexistence between tree species, it does not cover other sessile plant species that can grow with trees under their canopies covered by the lattice system. Moreover, though the mentioned model showed coexistence of tree species by varying the seedling establishment rate, it is clear that in the long run, the model will lead to competitive exclusion similar to other lottery models. However, comparing this to our results, the lattice system presented here showed a more stable coexistence in longer periods (Figs 1a, 2a and 3a, S2 and S3a–c).

In addition, species-specific local variability may also be implemented into mortality rates. The mortality variability alone guarantees the coexistence of many species (Fig. 1e). When the basal mortality *D* is decreased, the number of surviving species increases significantly, indicating the avoidance of extinction. In contrast, the effect of the fluctuation width *h* of mortality rate behaves quite different. When *h* is close to zero, the species with the highest birth rate will instantly win. However, when *h* is slightly increased, the number of coexisting species rapidly increased reaching an optimum value for *h*. This slight increase has an “equalizing effect” among species reducing the advantage of superior species. Nevertheless, when *h* is further increased, the number of survivors decreases gradually because the average mortality of all species increases with an increase in *h* (Fig. 1e). Furthermore, combined variabilities in reproduction and mortality improves system diversity (Fig. 1f).

Here the variability should be simultaneously species-specific and site-specific; otherwise almost all species are excluded by competition (Table 1, Figs 2b–d and 3b–d). This variability preserves tradeoffs between species, providing each one a number of sites where they are advantageous over other species (Fig. 2a). This species-specific site variability is preserved even if the number of competing species is increased. This indicates that the coexistence of multiple species is guaranteed by spatial heterogeneity.

Traditional mathematical models of coexistence have dealt with mostly two or three species. Actually, mathematical analysis become extremely cumbersome and almost impossible because of the number of parameters, e.g., birth rates, mortality rates of each species and competitive coefficients among species. Almost no models dealing with 5 or more species are investigated in the previous modeling of competition. However, in natural terrestrial plant communities, we find at least 10 or more species, conform to a study by Takenaka where coexistence between trees was possible and affected by establishment rate^14^. To clarify, our model is not a simple multi-species expansion of the Lotka-Volterra competition model (LVCM), where coexistence is only possible if interspecific competition is weaker than intraspecific competition. The basic model used here (Controls 1, 2 and 3 in Table 1) is the LVCM where all interspecific and intraspecific competitions are unity (i.e., the competitive coefficients *a*_*ij*_ = *a*_*ii*_ = 1 for all species *i*,*j*)^15^. Here, in Controls 1 and 2, the species with the highest basic birth rate (*B*_*i*_) tends to win, excluding all other species, while in Control 3 the species with the real highest birth rate is determined by the product of the basic birth rate and the species randomness, i.e., *B*_*i*_*ε*_*i*_ (Equation 3). Only when both species and site randomness are combined, superiority of species varies depending on the site, allowing the coexistence of various species (Table 1).

We should note that the coexistence of two or three species must be qualitatively different from that of 10 or more species. For example, while two species can coexist in a given range of parameter combinations, the coexistence of 3 species becomes much narrower in the parameter combinations. By adding one more species, the tradeoff condition that allows the coexistence of all species becomes tighter. Given 10 or more species, the coexistence region (if it exists) becomes extremely minute. This is because coexistence is harder to achieve given ten species with more tradeoffs, compared to those with two or three species only.

The effect of spatial heterogeneity has been dealt with two-species system under stochastic fluctuation in aquatic ecosystems^15^,^16^,^19^,^21^. For example, in one model setting, temporal fluctuation in birth process promotes coexistence^16^, while in the other, temporal fluctuation in mortality promotes coexistence^19^. Thus various detail settings of growth and mortality has reached various outcomes. In the current model of terrestrial plant communities, the coexistence is promoted either by the birth process and/or by the death process which is more stable under longer ecological time scale and can accommodate more species.

Tilman proposed that the coexistence of many species in plant communities should be achieved by the physiological differences among species in microhabitats^1^,^2^ as there are differences in soil and light conditions among species^4^,^22^. As mentioned before, the heterogeneity of soil conditions is high in almost all plant communities^20^,^23^, but the implementation of microhabitat heterogeneity has never been achieved due to mathematical difficulties. By the modification of lattice models, we could implement species-specific locality, where each site is assigned by a species-specific probability or parameter affecting birth or death rate.

Tilman also proposed the Lotka-Volterra competition model with tradeoffs in survival and dispersal^24^. One example is given *n* species, competitive coefficients are ordered from strongest to weakest (e.g., S_1_ > S_2_ > S_3_ > … > S_n_) but the colonization ability is the opposite. That is, weaker species can always occupy vacant sites where stronger species cannot invade. This model is another possibility to explain coexistence in plant communities in general. However, as Tilman suggested, many species can only coexist in very rare situations in this model.

In the traditional model, plant seedlings can successfully settle on a site depending on the probability of settlement rates. Therefore, successful settlement becomes random. In the current model, the seedling settlement also looks random. However, if we focus on one specific site, a certain species almost always occupies (settles) it. All species have the advantage of settlement over other species in a certain number of sites. As a matter of fact, it is this site advantage that guarantees the coexistence of multiple species.

The species-specific microhabitat locality introduced in the current model should be easily expected from the heterogeneity of soil conditions, e.g., nutrition, water content, minerals, fungi, and bacteria^20^,^23^. The heterogeneity of these conditions should affect the growth and survival of seedlings differently depending on the needs of each species. However, it is the extent to which the soil microhabitat will affect the number of coexisting species that remains an important question. Our results should be sufficient to demonstrate the coexistence of at least 10 species in terrestrial plant communities, seen that, limiting the dispersal of seeds locally increases the number of surviving species based on the simulation results (Fig. 4b). This can be the effect of limited competition between species since local reproduction is a form of avoidance. Overall, given the microhabitat and species-specific differences, diversity is preserved with at least 10 species surviving for any dispersal distance tested.

One of the merits of this model is its wide applicability to various communities and ecosystems. The current model can be applied to many terrestrial communities including forests, grasslands and wetlands. It should be also applicable to soil biota in general, aquatic plant and animal communities in tidal zones and marine benthos. By some modifications of lattice setting, it may be applicable to terrestrial faunal communities, such as vertebrates and insects. Here species-specific locality is the key factor to maintain the coexistence of multiple species. Thus, this model can capture the basic principle for the coexistence of species in communities and ecosystems.

## Methods

### Lattice Model

We consider a competitive system of multiple plant species (*i* = 1, 2, …, *s*, where *s* is the number of species). We apply a two-dimensional lattice (200 × 200), since plants compete for space (i.e., direct sunlight and soil). Each lattice site is either occupied by one and only one individual *X*_*i*_ of species *i* or empty (*O*). Overall dynamics are multi-species contact processes such that:


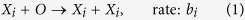



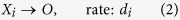


where the parameters *b*_*i*_ and *d*_*i*_, denote the birth and death rates, respectively. The simulation is carried out according to global interaction occurs where individual offspring can occupy any vacant lattice site in the lattice system.

In this lattice model, all species (*i* = 1, 2, …, *s*) compete for a vacant space and the outcomes depends solely on the growth rates of a species in each site.

We now introduce the site- and/or species- specificity in the birth rate *b*_*i*_, such that


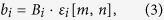


where *B*_*i*_ is the fecundity of species *i* and *ε*_*i*_[*m*, *n*] is the random parameter representing the specificity of species *i* at site [*m*, *n*]. In order to reach the stable state quickly, we introduce the variation (differences) in the basic fecundity *B*_*i*_ among species, such that B_i_ = *a* − (*i* − 1)*r* for *i* = 1, 2, …, *s*. Here, we set the minimum difference between species birth rate 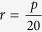, where *p* is an upper bound for the difference between birth rates. Note that the birth rate of the most superior species is *B*_1_ = *a* and the exact difference between the birth rates of the most and least superior species becomes 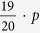.

We can consider *B*_*i*_ and *ε*_*i*_[*m*, *n*] as the species-specific fecundity and the local settlement rate, respectively. The random parameter *ε*_*i*_[*m*, *n*] follows a standard uniform distribution over [0, 1] with 32,767 divisions. Note that, if there is no species specificity, *ε*_*i*_[*m*, *n*] = *ε*[*m*, *n*] and if there is no site specificity, *ε*_*i*_[*m*, *n*] = *ε*_*i*_. The mortality rate is kept constant at *d*_1_ = 0.1 for all species in the entire lattice space for all simulations, except some extended models.

In the extended model, we introduce the random parameter *δ*_*i*_[*m*, *n*] into the death rate as follows:


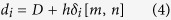


where *D* is the basal (lowest) mortality of all species at the entire lattice space, *h* is the range of mortality differences and *δ*_*i*_[*m*, *n*] is the random parameter *δ* representing the specificity of species *i* and site [*m*, *n*]. Here the death rates varies between *D* and *D* + *h*, since site- and species-specific death rates cannot be zero (“immortal”) or very close to zero.

### Simulation procedure

The simulation procedures of global interaction are as follows:

1. Plant species cells are distributed randomly over some of the square-lattice points along the initial density *I*_*i*_ in such a way that each point is occupied by one and only one individual of a certain species, if the point is occupied.
2. The reaction processes are performed in the following manner.
  - To perform the single body reaction or the death process (2), choose one square-lattice point randomly. If the point is occupied by an individual *X*_*i*_, then change it to *O* with probability *d*_*i*_. No change otherwise.
  - Next, perform the two-body reaction or the birth process (1) by selecting two lattice points randomly. If the selected pair are *X*_*i*_ and *O*, respectively, then the latter point will become *X*_*i*_ with probability *b*_*i*_. Otherwise, the points remain unchanged. Here, we utilize periodic boundary conditions.
3. Repeat step 2 *L* × *L* times, where *L* × *L* is the total number of the square-lattice sites. Here we set *L* = 200. This step is called a Monte Carlo step.
4. Repeat step 3 for a specific number of Monte Carlo steps.

The simulation procedure for local dynamics is similar except for the two-body reaction process. Two adjacent lattice sites are randomly selected rather than selecting them complete at random.

## Additional Information

**How to cite this article**: Tubay, J. M. *et al*. Microhabitat locality allows multi-species coexistence in terrestrial plant communities. *Sci. Rep.* **5**, 15376; doi: 10.1038/srep15376 (2015).

## Supplementary Material

Supplementary Information

## Figures and Tables

**Table 1:** Coexistence of 2 species at 5,000 steps in a lattice Lotka-Volterra competition model with species- and site-specific variability in birth rates and its three controls: (1) no variability, (2) site-specific variability only, and (3) species-specific variability only.

Model	Variability	Results			
	Species/Site	None	Birth only	Death only	Both
	Yes/Yes	–	Coexist	Coexist	Coexist
Control 1	No/No	Exclude	–	–	–
Control 2	No/Yes	–	Exclude	Exclude	Exclude
Control 3	Yes/No	–	Exclude*	Exclude*	Exclude*

*Number of surviving species depends on the trials.
