# Supplementary material for: Microhabitat locality allows multi-species coexistence in terrestrial plant communities
Source: Sci Rep. 2015 Oct 20;5:15376. doi: 10.1038/srep15376 (PMC4613359; doi:10.1038/srep15376)
Supplement: Supplementary Information [file srep15376-s1.pdf]

Microhabitat locality allows multi-species coexistence in terrestrial plant communities

Jerrold M. Tubay[1,2], Keisuke Suzuki[3], Takashi Uehara[1], Satoshi Kakishima[1], Hiromu Ito[1], Atsushi Ishida[4], Katsuhiko Yoshida[5], Shigeta Mori[6], Jomar F. Rabajante[1,2], Satoru Morita[3], Masayuki Yokozawa[1], Jin Yoshimura[1,3,7,8,*]

SUPPLEMENTARY FIGURES

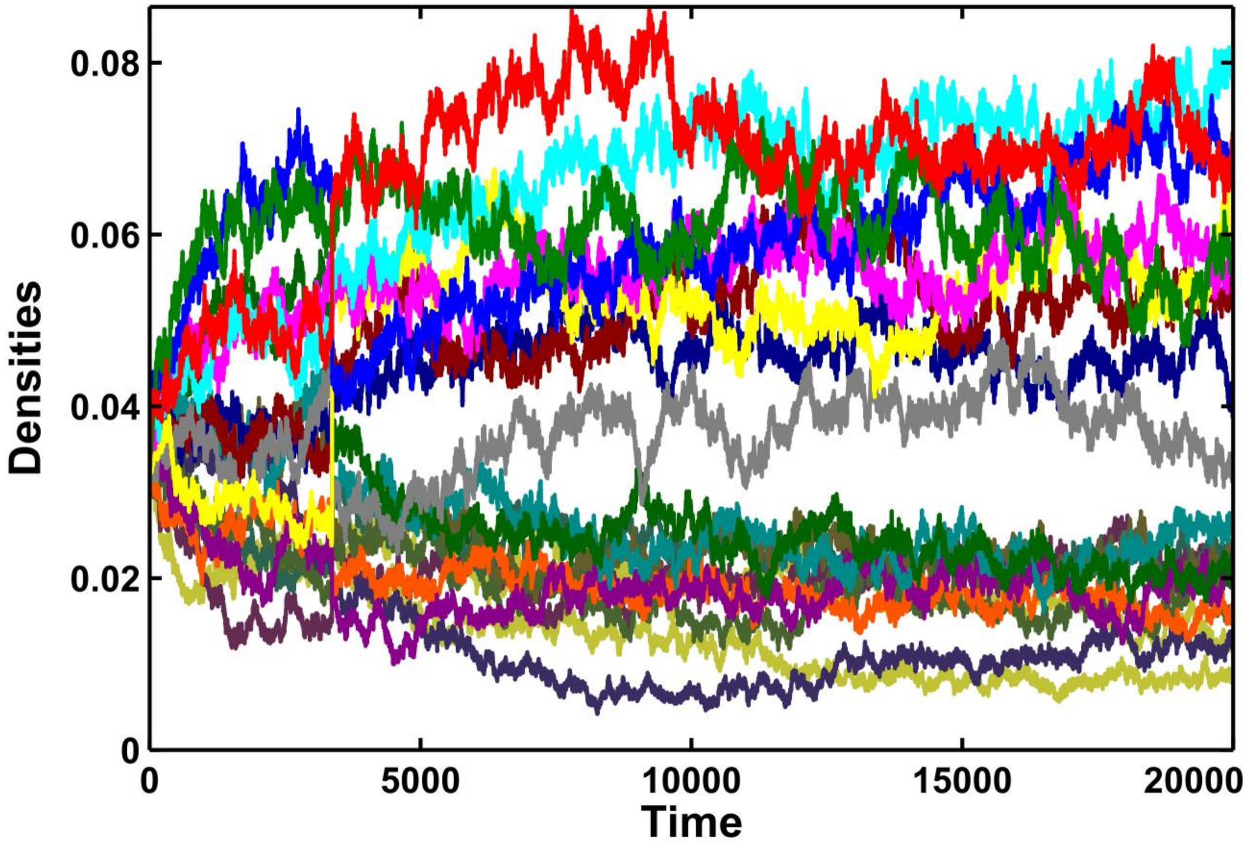

Figure S1. Population local dynamics of 20-species community in a lattice Lotka-Volterra competition model with species- and site-specific variability.

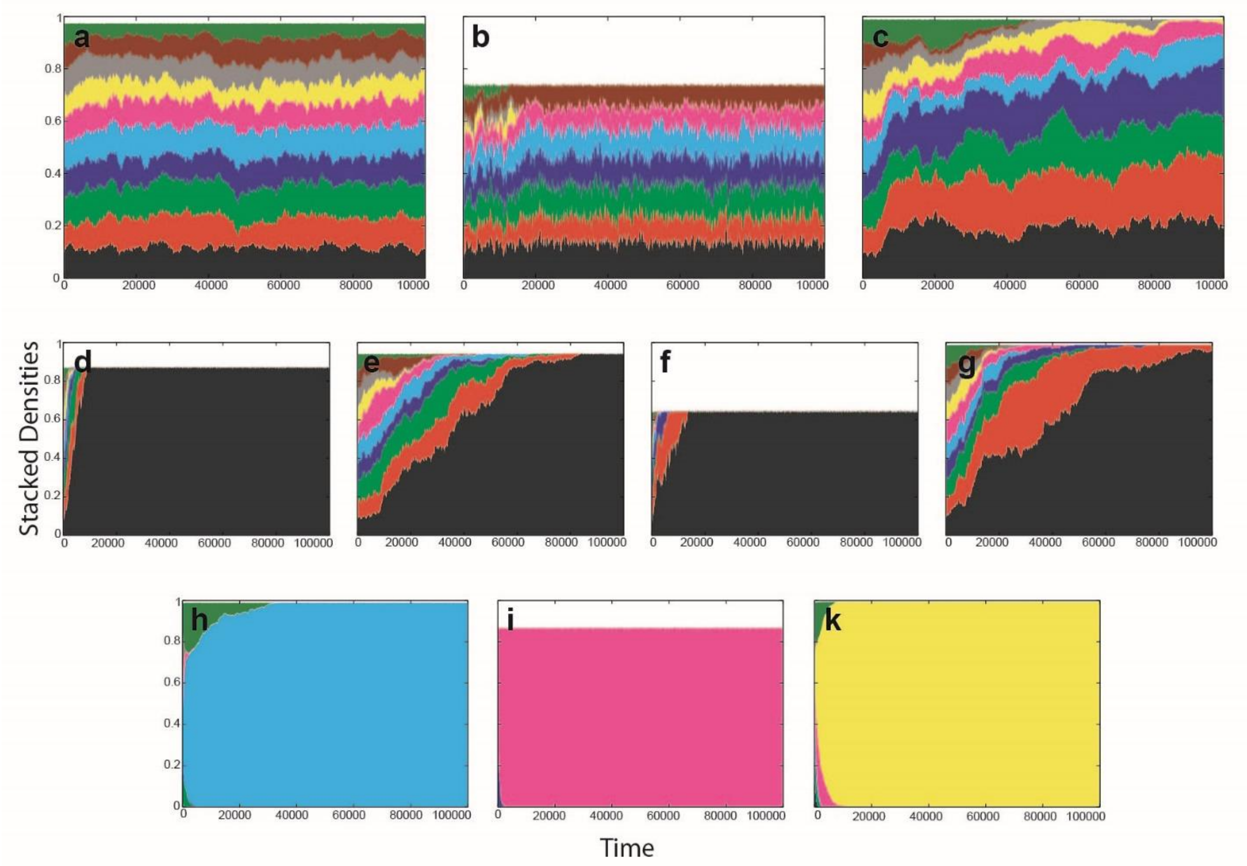

Figure S2. Stacked densities of 10 species at 100,000 steps in a lattice Lotka-Volterra competition model with and without species- and site-specific variability: (**a**) with species- and site-specific variability for both birth and death rates, (**b**) with species- and site-specific variability for birth rates only, (**c**) with species- and site-specific variability for death rates only, (**d**) without species- and site-specific variability, (**e**) with site-specific variability only for both birth and death rates (**f**) with site-specific variability only for birth rates (**g**) with site-specific variability only for death rates (**h**) with species-specific variability only for both birth and death rates (**i**) with site-specific variability only for both birth rates (**j**) with site-specific variability only for both death rates.

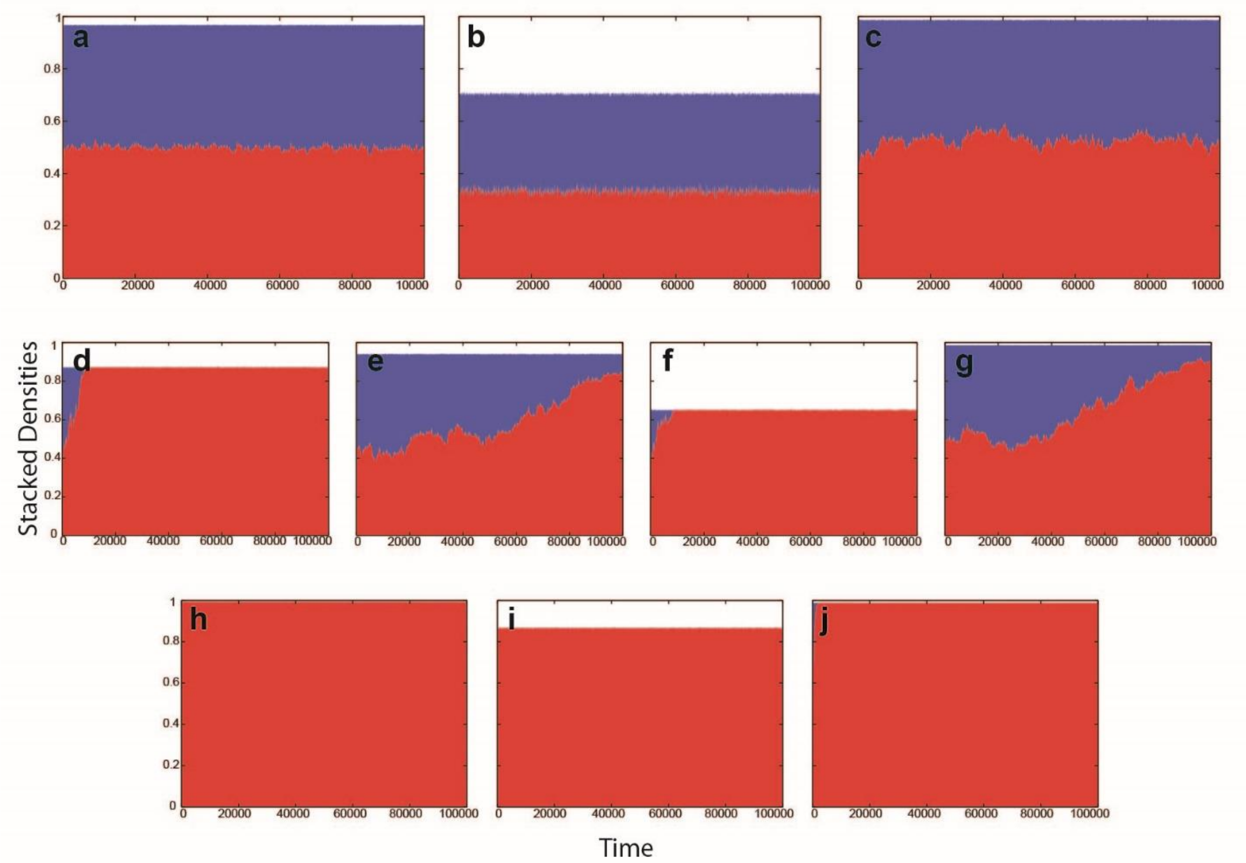

Figure S3. Stacked densities of 2 species at 100,000 steps in a lattice Lotka-Volterra competition model with and without species- and site-specific variability: (**a**) with species- and site-specific variability for both birth and death rates, (**b**) with species- and site-specific variability for birth rates only, (**c**) with species- and site-specific variability for death rates only, (**d**) without species- and site-specific variability, (**e**) with site-specific variability only for both birth and death rates (**f**) with site-specific variability only for birth rates (**g**) with site-specific variability only for death rates (**h**) with species-specific variability only for both birth and death rates (**i**) with site-specific variability only for both birth rates (**j**) with site-specific variability only for both death rates.
